# Reliability and validity of the Chinese version of the Brief Emotion and Regulation Beliefs Scale in Chinese nursing students

**DOI:** 10.1186/s12912-022-00992-1

**Published:** 2022-08-09

**Authors:** Dongmei Zhang, Liu Yang, Congzhi Wang, Ting Yuan, Huanhuan Wei, Jing Li, Yunxiao Lei, Lu Sun, Xiaoping Li, Ying Hua, Mingming Liu, Haiyang Liu, Lin Zhang

**Affiliations:** 1 Department of Pediatric Nursing, School of Nursing, Wannan Medical College, 22 Wenchang West Road, Higher Education Park, An Hui Province, Wuhu City, People’s Republic of China; 2 Department of Internal Medicine Nursing, School of Nursing, Wannan Medical College, 22 Wenchang West Road, Higher Education Park, An Hui Province, Wuhu City, People’s Republic of China; 3 Obstetrics and Gynecology Nursing, School of Nursing, Wannan Medical College, 22 Wenchang West Road, Higher Education Park, An Hui Province, Wuhu City, People’s Republic of China; 4 Department of Surgical Nursing, School of Nursing, Wannan Medical College, 22 Wenchang West Road, Higher Education Park, An Hui Province, Wuhu City, People’s Republic of China; 5 Department of Emergency and Critical Care Nursing, School of Nursing, Wannan Medical College, 22 Wenchang West Road, Higher Education Park, An Hui Province, Wuhu City, People’s Republic of China; 6 Rehabilitation Nursing, School of Nursing, Wanna Medical College, 22 Wenchang West Road, Higher Education Park, An Hui Province, Wuhu City, People’s Republic of China; 7 Student Health Center, Wannan Medical College, 22 Wenchang West Road, Higher Education Park, An Hui Province, Wuhu City, People’s Republic of China

**Keywords:** Emotions, Nursing, Students, Reliability, Validity

## Abstract

**Background:**

Nursing students are experiencing complex learning environments and will experience complex work environments in future clinical work, which lead to emotional problems easily. However, one’s beliefs about controlling their emotions portend a series of vital psychological outcomes. So, it is especially important to search for suitable tools to assess the emotion and regulation beliefs of nursing students and give timely intervention to improve their physical and mental health. This study aimed to translate the American version of the Emotion and Regulation Beliefs Scale (ERBS) into Chinese, revise the original scale and form a simplified version, and assess the reliability and validity of the brief Chinese version in nursing students.

**Methods:**

The study adopted a cross-sectional design and the multistage sampling design. The ERBS was translated into Chinese, and the reliability and validity of the Chinese version were tested in 980 nursing students.

**Results:**

The content validity index was 0.920. Exploratory factor analysis supported a three-factor model for the Chinese version of Brief**-**ERBS, and confirmatory factor analysis indicated that the model fit the Brief**-**ERBS well. Furthermore, the three-factors model was obtained by using exploratory factor analysis, explaining 51.023% variance, and the communalities of the items ranged from 0.359 to 0.680. With modified confirmatory factor analysis, the fit indices were chi-square/degree of freedom (CMIN/DF) = 4.092, goodness of fit index (GFI) = 0.949, adjusted goodness of fit index (AGFI) = 0.927, comparative fit index (CFI) = 0.913, incremental fit index (IFI) = 0.914, Tucker Lewis index (TLI) = 0.908, root-mean-square error of approximation (RMSEA) = 0.061. The two-tailed independent samples t-test showed the scores of the top (50%) and low (50%) groups reached the level of significance (*P* < 0.001). A highly positive correlation between the Brief-ERBS total score and the ERBS total score was found (*r* = 0.972, *P* < 0.01). The Cronbach’s α coefficient of the scale was 0.798, the split-half reliability coefficient was 0.784, and the retest coefficient was 0.879.

**Conclusion:**

The Chinese version of Brief**-**ERBS has good reliability and validity, and may be used for the beliefs about emotional management in Chinese nursing students.

**Supplementary Information:**

The online version contains supplementary material available at 10.1186/s12912-022-00992-1.

## Background

Emotion regulation processes are goal-oriented behaviors functioning to modify dynamic features of emotion, such as the magnitude and duration of behavioral, experiential, and physiological responses [1, 2]. Emotion regulation refers to the processes by which we influence which emotions we have, when we have them, and how we experience and express them. It could be affected by intra- and extra-familial social factors [3], and also improved by interventions [4]. Emotion dysregulation has been linked to a variety of mental problems [5, 6], and teens with difficulty modulating emotions may be involved in more illegal activities, for poorly regulated emotions may interfere with cognitive function that reminds youth of rules during decision making [7]. Conversely, effective emotion regulation promotes mental health and is related to multiple positive mental outcomes, such as greater perceived well-being, better interpersonal relationships, and better physical health [5]. As a result, emotion regulation is crucial to successful social interactions and health and is used a lot in our daily life [8–10].

Nursing students are facing a range of pressure, such as academic pressure, interpersonal pressure, and professional pressure, which cause emotional issues easily. Nursing education involves situations where students engage in interaction with other people, learning to care for and help patients involves a variety of emotions [11]. For example, dissection and autopsies in the course of the preclinical study can provoke strong emotional reactions in some medical students [12]. The clinical placement experiences can elicit negative emotions in nursing students. However, nursing students may be unprepared for regulating their emotions [13–15]. Emotion is a key source of stress for the early career of nurses and nursing students. Developing emotional intelligence behaviors should be a very useful measure to improve academic and clinical performance [16–18], and effective emotion regulation to enhance nursing students’ professional identity and build the skills for effective patient care and their health and well-being [19, 20]. In clinical work, nurses do a lot of physical and mental work, but also pay more emotional work [21, 22]. In recent years, more and more scholars have realized the importance of nurses' physical and mental health to guarantee clinical nursing quality and stabilize nursing teams [21, 23, 24]. As future nurses, nursing students are experiencing complex learning environments and will experience complex work environments in future clinical work, which probably lead to emotional problems directly [25, 26]. However, one’s beliefs about controlling their emotions portend a series of vital psychological outcomes [27]. So, it is especially important to search for suitable tools to assess the emotion and regulation beliefs of nursing students, and give timely intervention to improve their physical and mental health [10, 27].

The Emotion and Regulation Beliefs Scale (ERBS), which was originally developed by Veilleux et al. in America, is a simple and effective tool to assess emotion and regulation belief. The ERBS assesses beliefs that emotions can hijack self-control, emotion regulation is a worthwhile pursuit, and emotions can constrain behavior. The ERBS has excellent internal consistency and powerfully predicts clinically relevant outcomes even after controlling for an existing short measure of beliefs in emotion controllability [28]. In Veilleux et al. 's study, participants were recruited from Amazon Mechanical Turk, has showed that negative beliefs about emotion are higher in individuals who would meet criteria for an eating disorder and depression, and Emotion Constraint predicts clinical groups uniquely [28].

Therefore, this study was to translate the American version of the ERBS into Chinese, revise the original scale and form a simplified version, and assess the reliability and validity of the brief Chinese version in nursing students.

## Methods

### Design and sample

A cross‐sectional survey was conducted from June to December 2015 in the Liaoning Province, China. The participants were nursing students in Dalian University and Jinzhou Medical University. All participants completed the tests voluntarily. Approval for the study was obtained from the College of Nursing’s Research Committee at Wannan Medical College (2,015,003). The inclusion criteria used for the participants were as follows: (i) full-time nursing student on campus; (ii) able to communicate and read; and (iii) consent to participate. Based on the criterion proposed by Kendall (10 to 20‐fold the number of items and expanded by at least 10% to ensure a sufficient sample size) [29], a sample size of at least 231 was calculated since the number of items in ERBS is 21. Finally, A total of 1087 nursing students took part in the survey, and 980 students completed the questionnaires effectively. 30 randomly selected students were invited to complete the questionnaires again two weeks later to test the reliability of the retest.

### The instrument

The ERBS is a 21-item, three-factors scale, with factor one (emotion constraint, nine items), factor two (regulation worth, seven items), and factor three (Hijack, five items) [28]. Factor one represents the belief that emotions are forces that constrain or narrow an individual’s choices in an emotional situation, factor two represents the belief that emotion regulation is both possible and worthwhile, and factor three represents the belief that emotions hijack or usurp peoples’ self-control. The ERBS uses a 5-point Likert scale with response choices ranging from “very disagree” to “very agree”. The score of the scale ranges from 21 to 105. The higher the score of regulation worth represents the higher the emotion and regulation beliefs. Conversely, the higher the score of the emotion constraint and Hijack represents the weaker the emotion and regulation beliefs.

### Translation procedure

Several steps were undertaken by translation guidelines [30, 31]. First, two bilingual professional translators translated the ERBS from English into Chinese. Another two bilingual professional translators translated the Chinese version back into English. Second, a bilingual expert panel consisting of four nursing experts and two psychology experts evaluated the cultural and linguistic equivalence of each item. Third, a preliminary field test was conducted on 30 nursing students with the Chinese version. According to their feedback, the ERBS was revised. The ERBS of English and Chinese versions are shown in Table 1.

**Table 1:** The emotion and regulation beliefs scale (English version and Chinese version)

Item	Item content (English/Chinese)	Score
Q1	Emotions operate like a floodgate that is either open or closed. In other words, emotions are either “on” or “off.”	1 2 3 4 5
	情绪控制就像水闸门一样或开或关, 即情绪要么有, 要么没有	
Q2	Emotions can either be expressed entirely or hidden from others-it isn’t possible to share only part of an emotional response	1 2 3 4 5
	情感只能完全表达或隐藏, 不可能同别人部分分享	
Q3	People can learn to control/regulate their emotions	1 2 3 4 5
	人们能够学习控制或调节自己的情绪	
Q4	People are ruled by their emotions	1 2 3 4 5
	人们被自己的情绪所左右	
Q5	Putting forth effort to alter emotional experience is valuable	1 2 3 4 5
	努力改变情绪经历过程是值得追求的	
Q6	When a person has a strong emotional reaction to another person, they will always feel that way about that other person	1 2 3 4 5
	当一个人对另一个人有很强的情感回应的时候, 他们通常会想法一致?	
Q7	When people are feeling down, they have to wait for a better mood to arrive before they can be productive	1 2 3 4 5
	当人们感觉情绪低落的时候, 他们必须等到情绪好的时候才能达到之前工作效率的水平	
Q8	People would be better off if they took time to figure out where their emotions come from	1 2 3 4 5
	人们如果能知道自己情绪的出处就更好了	
Q9	When strong emotions are present, they dictate what a person says or does	1 2 3 4 5
	当一个人强烈的情绪出现时, 这个人的语言或行为就被这种情绪所控制着	
Q10	When an emotion comes along, it will continue unless there is a change in the environment	1 2 3 4 5
	当情绪产生时, 它会不断持续直到周围的环境发生了变化	
Q11	When people acknowledge their emotions, the emotions will completely take them over	1 2 3 4 5
	当人们表露自己的情绪的时候, 他们完全被这种情绪所控制	
Q12	Learning how to alter strong emotions is a worthwhile pursuit	1 2 3 4 5
	学习如何改变强烈的情绪是一个值得追求的事	
Q13	It is possible, with effort, to alter strong feelings in any situation	1 2 3 4 5
	无论在何种情况下, 通过努力改变强烈的情绪都是可能的	
Q14	When a person feels really angry, it’s virtually impossible to not take the anger out on people or objects nearby	1 2 3 4 5
	当一个人感到非常愤怒时, 几乎不可能不把愤怒发泄在周围的人或物上	
Q15	People are slaves to their emotions	1 2 3 4 5
	人是情绪的奴隶	
Q16	People would be better off if they spent more time learning how to control their emotions	1 2 3 4 5
	人们如果能花费更多的时间去学习如何控制自己的情绪就更好	
Q17	Strong emotions will make people do things they wouldn’t normally do	1 2 3 4 5
	强烈的情绪会使人做平常不会做的事情	
Q18	When feelings of sadness take over, a person can’t really do anything but wallow in the misery	1 2 3 4 5
	当悲伤占据一个人的情感时, 一个人除了沉溺在痛苦之中, 将无法做任何事	
Q19	People benefit from learning how to regulate their feelings	1 2 3 4 5
	人们从如何控制自己的情绪中获益	
Q20	It’s virtually impossible for people to act opposite to the way they feel	1 2 3 4 5
	人们实际上很难违背自己的感情行事	
Q21	Emotions make people lose control	1 2 3 4 5
	情绪让人失控	

### Data collection

The questionnaire consisted of the Chinese version of the ERBS, as well as socio‐demographic information. The multistage sampling design was used to conduct this study. Firstly, Dalian University and Jinzhou Medical University were randomly selected from 6 nursing colleges in Liaoning Province. Secondly, 50% classes (ranging from one to three classes) in each grade were randomly selected from both universities [32], including the second batch of undergraduate, the third batch of undergraduate and higher vocational schools. As a result, a total of 39 classes were selected in the two universities. These included 24 classes in Dalian University and 15 classes in Jinzhou Medical University. Thirdly, the all of students ranged from 25 to 30 in each class were selected by cluster sampling. As a result, a total of 1125 nursing students were selected, including 694 nursing students in Dalian University and 431 nursing students in Jinzhou Medical University. The nursing students were interviewed face to face by the trained interviewers. Before data collection, 9 postgraduates in charge of the interview received unified training to learn how to use standardized language and instructions. Finally, of 1125 nursing students, a total of 1087 took part in the survey, and 980 completed the questionnaires effectively, including 640 and 340 in Dalian University and Jinzhou Medical University respectively. And the average time to finish the survey questionnaire was about 3.5 min.

### Statistical analysis

SPSS 20.0 and AMOS 22.0 were used to analyze the data. Pearson’s correlation analysis was used to advise the item to be removed or not. Content validity index (CVI) was used to evaluate the content validity of the Brief-ERBS, and 5 relevant experts were invited to assess the content validity. The exploratory factor analysis (EFA) and confirmatory factor analysis (CFA) were used to measure the construct validity. Discriminant validity was analyzed by using a two-tailed independent samples t-test. Convergent validity was assessed by correlation between the Brief-ERBS and the ERBS. And the reliability of the Brief-ERBS was calculated by Cronbach’s alpha, split-half reliability, item-total score correlations, and retest reliability.

### Ethical considerations

All individuals have provided informed consent before the data collection. Approval for the study was obtained from the College of Nursing’s Research Committee at Wannan Medical College (2,015,003).

## Results

### The sample

Of the participating 980 nursing students, their ages ranged from 17 to 26 years, most of them were female (871, 88.88%). Table 2 shows the other demographic characteristics of the participators.

**Table 2:** Frequency distribution of demographic characteristics (*n* = 980)

**Variables**	Groups	N	**%/‾*X*** ± *S*
**City**	Jinzhou	340	34.69
	Dalian	640	65.31
**Sex**	Male	109	11.12
	Female	871	88.88
**Age (years)**	17–26	980	20.55 ± 1.45
**Grade**	Freshman	294	30.00
	Sophomore	412	42.04
	Junior	274	27.96
**Only child**	Yes	418	42.65
	No	562	57.35
**Education**	Higher vocational schools	301	30.71
	The third batch of undergraduate	183	18.67
	The second batch of undergraduate	496	50.61

### Items analysis

In the 21-item ERBS, there was statistically significant (*P* < 0.001) in item-total score correlations based on Pearson correlation analysis, and correlations ranged from 0.252 to 0.596 (Table 3). Three items (Q1, Q2, and Q6) were removed for item-total score correlations less than 0.4 [33]. However, the score of Q3 (people can learn to control/regulate their emotions) had significant positive correlation with total scores, and the correlation coefficient was 0.370, which was somewhat below 0.4. And Q3 was almost important and determined that this was a belief of emotion and regulation according to experts’ advice and the cultural background in China. Therefore, Q3 was retained for further testing. Consequently, 18-item ERBS were established. In the 18-item ERBS, item-total score correlations ranging from 0.420 to 0.613 except Q3 (*r* = 0.377), with higher correlation coefficients than the 21-item. Based on several EFA after that, both Q14 and Q9 had a lower load value (0.269 ~ 0.318 and 0.224 ~ 0.360) than 0.40 on all common factors and was removed [33], Q16 was deleted for in the fourth factor lonely which was too little item, Q20 was deleted for belonging to the Hijack which was not in according with original scale in the emotion constraint. Finally, the Chinese version of Brief**-**ERBS consisting of 14 items was formed.

**Table 3:** Item-total score person correlation analysis results in ERBS of 21 Items (*n* = 980, α = 0.05)

Item	Item content	*r*	*P*
Q1	Emotions operate like a floodgate that is either open or closed. In other words, emotions are either “on” or “off.”	0.350	< 0.001
Q2	Emotions can either be expressed entirely or hidden from others-it isn’t possible to share only part of an emotional response	0.252	< 0.001
Q3	People can learn to control/regulate their emotions	0.370	< 0.001
Q4	People are ruled by their emotions	0.490	< 0.001
Q5	Putting forth effort to alter emotional experience is valuable	0.512	< 0.001
Q6	When a person has a strong emotional reaction to another person, they will always feel that way about that other person	0.366	< 0.001
Q7	When people are feeling down, they have to wait for a better mood to arrive before they can be productive	0.539	< 0.001
Q8	People would be better off if they took time to figure out where their emotions come from	0.536	< 0.001
Q9	When strong emotions are present, they dictate what a person says or does	0.574	< 0.001
Q10	When an emotion comes along, it will continue unless there is a change in the environment	0.490	< 0.001
Q11	When people acknowledge their emotions, the emotions will completely take them over	0.540	< 0.001
Q12	Learning how to alter strong emotions is a worthwhile pursuit	0.586	< 0.001
Q13	It is possible, with effort, to alter strong feelings in any situation	0.476	< 0.001
Q14	When a person feels really angry, it’s virtually impossible to not take the anger out on people or objects nearby	0.541	< 0.001
Q15	People are slaves to their emotions	0.458	< 0.001
Q16	People would be better off if they spent more time learning how to control their emotions	0.532	< 0.001
Q17	Strong emotions will make people do things they wouldn’t normally do	0.579	< 0.001
Q18	When feelings of sadness take over, a person can’t really do anything but wallow in the misery	0.433	< 0.001
Q19	People benefit from learning how to regulate their feelings	0.504	< 0.001
Q20	It’s virtually impossible for people to act opposite to the way they feel	0.457	< 0.001
Q21	Emotions make people lose control	0.596	< 0.001

### Validity

#### Content validity

The CVI of the items ranged from 0.850 to 0.930, and the CVI of the Brief**-**ERBS was 0.920.

#### Construct validity

##### Exploratory factor analysis

A Kaiser–Meyer–Olkin (KMO) value of 0.891 and a Bartlett spherical test value of 4740.447 (*df* = 210,* P* < 0.001) in an EFA of 21-items ERBS, showed that the factor analysis was suitable [33]. Five common factors were extracted after maximum variance rotation and explained 51.421% of the total variance (Table 4). A KMO value of 0.862 and a Bartlett spherical test value of 3147.164 (*df* = 91, *P* < 0·001) in an EFA of the Brief-ERBS, showed that the factor analysis was also suitable [33]. And three common factors were extracted by maximum variance rotation, which explained 51.023% of the total variance (Table 5).

**Table 4:** Factor load and communalities of each item in ERBS of 21 Items (*n* = 980)

Item	F1	F2	F3	F4	F5	Communalities
Q13	0.748	0.131	-0.058	0.071	-0.020	0.585
Q12	0.730	0.079	0.194	0.130	0.028	0.595
Q16	0.702	0.231	0.088	-0.101	0.070	0.569
Q19	0.654	-0.107	0.348	0.093	-0.058	0.573
Q5	0.592	-0.128	0.224	0.321	0.102	0.530
Q8	0.518	0.155	0.065	0.432	-0.020	0.484
Q18	-0.034	0.678	0.079	0.042	0.166	0.496
Q11	0.146	0.658	0.124	0.118	0.128	0.500
Q10	0.006	0.608	0.089	0.305	0.139	0.490
Q15	0.058	0.523	0.402	-0.234	0.102	0.505
Q20	0.130	0.498	0.207	0.136	-0.150	0.349
Q4	0.002	0.113	0.659	0.181	0.151	0.503
Q14	0.300	0.159	0.574	-0.016	0.055	0.448
Q17	0.268	0.237	0.565	0.157	-0.088	0.480
Q21	0.180	0.497	0.534	-0.054	-0.000	0.567
Q6	0.117	0.105	-0.001	0.664	-0.029	0.467
Q9	0.085	0.277	0.438	0.495	0.018	0.520
Q7	0.121	0.416	0.139	0.460	0.157	0.444
Q3	0.376	-0.357	0.319	0.414	0.072	0.548
Q1	0.071	0.030	0.162	0.089	0.768	0.631
Q2	-0.014	0.246	-0.052	-0.047	0.739	0.612

F1 contained Q5, Q8, Q12, Q13, Q16, and Q 19, F2 contained Q10, Q11, Q15, Q18, and Q20, F3 contained Q4, Q14, Q17, and Q21, F4 contained Q3, Q7, Q6, and Q9, F5 contained Q1, and Q2

**Table 5:** Factor load and communalities of each item in Brief-ERBS (*n* = 980)

Item	F1	F2	F3	Communalities
Q5	0.728	0.091	0.043	0.540
Q12	0.722	0.189	0.104	0.567
Q19	0.713	0.161	-0.059	0.538
Q3	0.666	-0.036	-0.083	0.452
Q13	0.622	0.073	0.112	0.405
Q8	0.579	-0.008	0.356	0.462
Q15	-0.063	0.793	0.092	0.641
Q21	0.198	0.672	0.224	0.540
Q4	0.254	0.537	0.079	0.359
Q17	0.402	0.418	0.169	0.366
Q7	0.227	0.024	0.792	0.680
Q10	0.006	0.281	0.665	0.521
Q18	-0.155	0.473	0.518	0.516
Q11	0.055	0.467	0.502	0.473

F1 contained Q3, Q5, Q8, Q12, Q13, and Q19, F2 contained Q4, Q15, Q17, and Q21, F3 contained Q7, Q10, Q11, and Q18

##### Confirmatory factor analysis

**Fig. 1:**
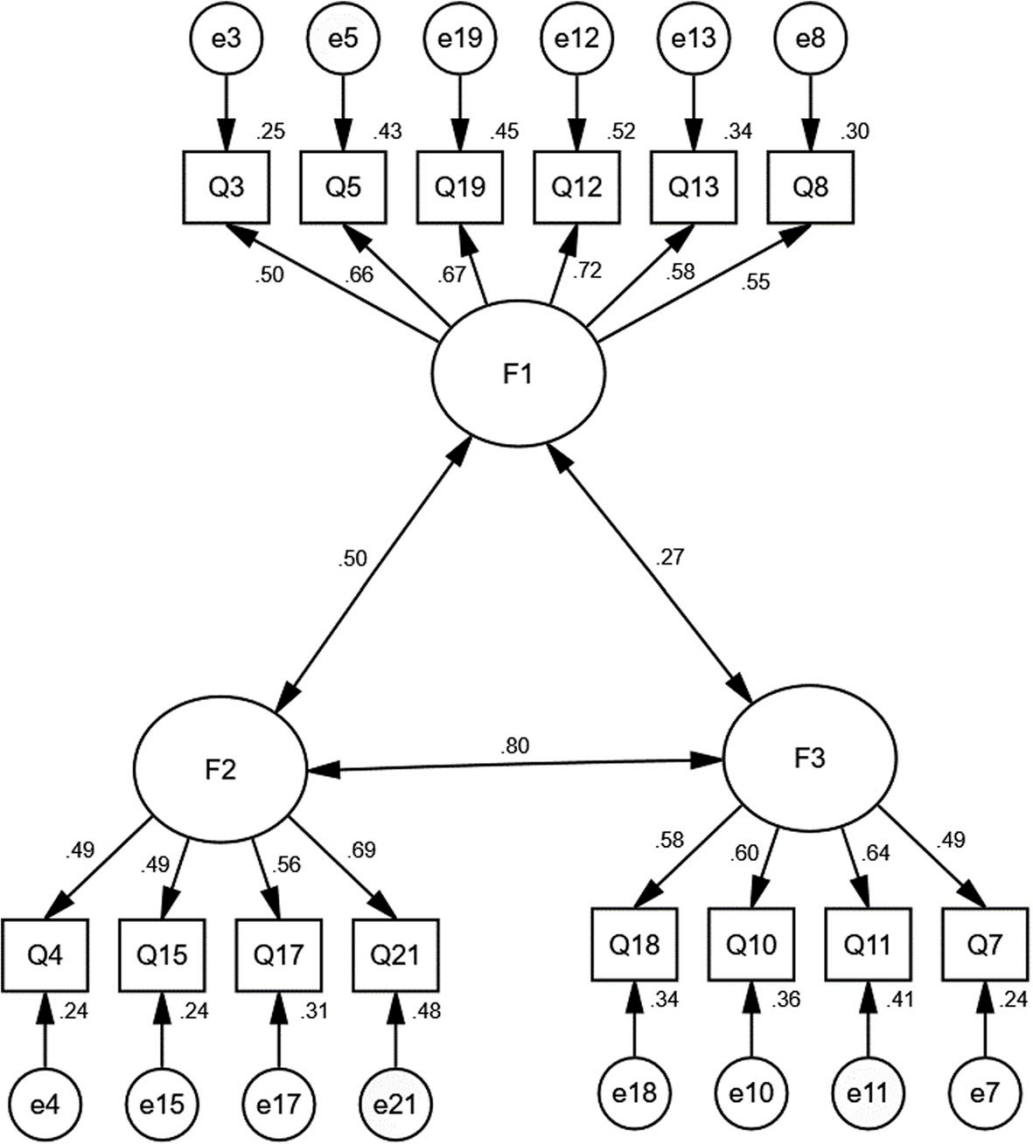
Standardized three-factors structural model of Brief-ERBS (*n* = 980); F1(Regulation Worth, six items), F2(Hijack, four items), and F3(Emotion Constraint, four items)

**Fig. 2:**
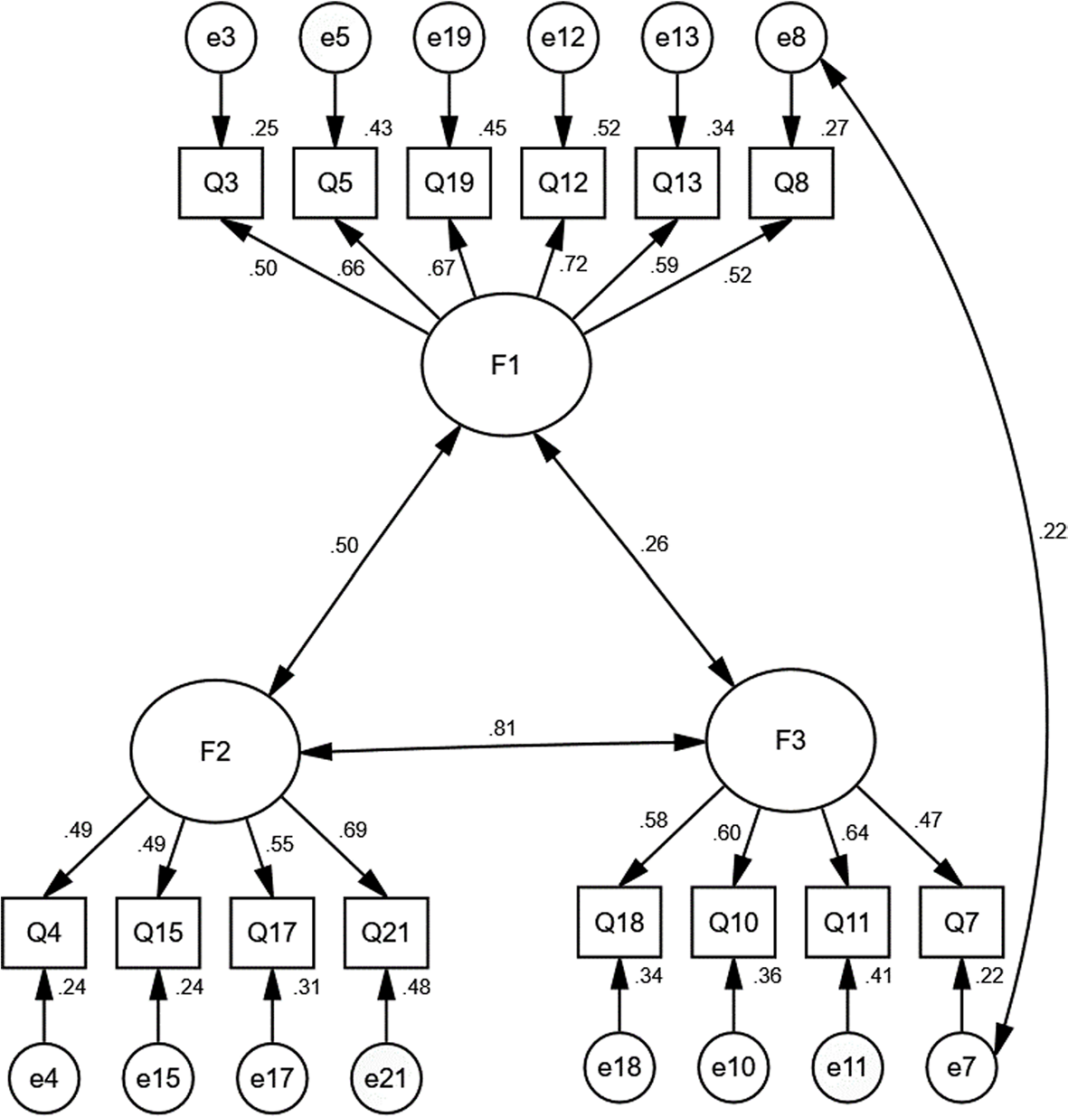
Standardized three-factors structural model of the modified Brief-ERBS (*n* = 980); F1(Regulation Worth, six items), F2(Hijack, four items), and F3(Emotion Constraint, four items)

With CFA, in an original three-factor model with the Brief**-**ERBS, the fit indices were not acceptable (Table 6 and Fig. 1). Then, modification indices were taken to improve the fit indices, and a new three-factors model was built and showed an acceptable goodness-of-fit, chi-square/degree of freedom (CMIN/DF) = 4.092, goodness of fit index (GFI) = 0.949, adjusted goodness of fit index (AGFI) = 0.927, comparative fit index (CFI) = 0.913, incremental fit index (IFI) = 0.914, Tucker Lewis index (TLI) = 0.908), root-mean-square error of approximation (RMSEA) = 0.061 (Table 6 and Fig. 2).

**Table 6:** Evaluation fitness of SEM model

Model	CMIN/DF	GFI	AGFI	NFI	RFI	IFI	TLI	CFI	PNFI	PCFI	RMSEA
Initial model	5.110	0.945	0.921	0.881	0.856	0.902	0.878	0.901	0.716	0.733	0.065
Modified model	4.092	0.949	0.927	0.907	0.900	0.914	0.908	0.913	0.716	0.733	0.061
Standard value	< 5.000	> 0.900	> 0.900	> 0.900	> 0.900	> 0.900	> 0.900	> 0.900	> 0.500	> 0.500	< 0.08

#### Discriminant validity

In our study, the scores of each item in the top (50%) and low (50%) groups were analyzed by using a two-tailed independent samples t-test. Table 7 shows that the score difference of each item in the 2 groups reached the level of significance (*P* < 0.001).

**Table 7:** Discriminant validity analysis in Brief-ERBS (*n* = 980)

**Item**	**Low-score group** **mean** ± ***SD***	**High-score group** **mean** ± ***SD***	***t***	***P***
Q3	2.99 ± 0.74	1.76 ± 0.43	32.235	< 0.001
Q4	3.46 ± 0.60	2.13 ± 0.54	36.550	< 0.001
Q5	2.96 ± 0.68	1.74 ± 0.44	33.512	< 0.001
Q7	3.54 ± 0.59	2.01 ± 0.52	42.921	< 0.001
Q8	2.93 ± 0.68	1.80 ± 0.40	32.087	< 0.001
Q10	3.64 ± 0.60	2.3 ± 0.56	36.161	< 0.001
Q11	3.66 ± 0.61	2.39 ± 0.58	33.347	< 0.001
Q12	2.87 ± 0.72	1.67 ± 0.47	31.029	< 0.001
Q13	3.07 ± 0.70	1.83 ± 0.37	34.582	< 0.001
Q15	4.01 ± 0.52	2.50 ± 0.62	41.172	< 0.001
Q17	3.04 ± 0.66	1.85 ± 0.36	35.286	< 0.001
Q18	4.08 ± 0.50	2.57 ± 0.57	44.154	< 0.001
Q19	2.83 ± 0.69	1.77 ± 0.42	28.988	< 0.001
Q21	3.40 ± 0.60	2.07 ± 0.51	37.260	< 0.001

#### Convergent validity

To evaluate the convergent validity of the Brief-ERBS in the context of our study, we examined the relationship between the Brief-ERBS and the ERBS. A highly positive correlation between the Brief-ERBS total score and the ERBS total score was found (*r* = 0.972, *P* < 0.01).

### Reliability

The Cronbach’s α of the Brief**-**ERBS was 0.798, the dimension of Regulation Worth was 0.782, the dimension of Hijack was 0.666, and the dimension of Emotion Constraint was 0.633. The split‐half reliability was 0.784. As seen in Table 8, there was a positive correlation and statistically significant (*P* < 0.001) in item-total score correlations, the correlations ranged from 0.400 to 0.634. The intragroup correlation coefficient was used to assess the retest reliability of the Brief**-**ERBS, and the retest coefficient was 0.879.

**Table 8:** Item-total score person correlation analysis results in Brief-ERBS (*n* = 980, α = 0.05)

Item	Item content	*r*	*P*
Q3	People can learn to control/regulate their emotions	0.405	< 0.001
Q4	People are ruled by their emotions	0.487	< 0.001
Q5	Putting forth effort to alter emotional experience is valuable	0.549	< 0.001
Q7	When people are feeling down, they have to wait for a better mood to arrive before they can be productive	0.528	< 0.001
Q8	People would be better off if they took time to figure out where their emotions come from	0.569	< 0.001
Q10	When an emotion comes along, it will continue unless there is a change in the environment	0.469	< 0.001
Q11	When people acknowledge their emotions, the emotions will completely take them over	0.521	< 0.001
Q12	Learning how to alter strong emotions is a worthwhile pursuit	0.634	< 0.001
Q13	It is possible, with effort, to alter strong feelings in any situation	0.525	< 0.001
Q15	People are slaves to their emotions	0.400	< 0.001
Q17	Strong emotions will make people do things they wouldn’t normally do	0.595	< 0.001
Q18	When feelings of sadness take over, a person can’t really do anything but wallow in the misery	0.409	< 0.001
Q19	People benefit from learning how to regulate their feelings	0.559	< 0.001
Q21	Emotions make people lose control	0.598	< 0.001

## Discussion

The testing results provide evidence that the Chinese version of the Brief**-**ERBS has good psychometric properties, and is a reliable and valid instrument. Therefore, the scale can be used for the beliefs about emotional management in Chinese nursing students.

The CVI of the items ranged from 0.850 to 0.930, and the CVI of the scale was 0.920, which indicated excellent content validity.

Construct validity was tested by EFA and CFA. The three factors the Brief**-**ERBS can explain 51.023% of the variation, which was significantly higher than the American version (36%), and similar to the five factors of the 21-item Chinese version (51.421%). However, the three factors (14 items) were simpler and proper than the five factors (21 items), which were simplified from the five factors (21 items) based on Pearson correlation analysis and several EFA.

Construct validity is usually assessed by factor analysis. The ideal result is that each item has a higher load value than 0.40 on one of the common factors, having a low load value on other common factors, and the cumulative variance contribution ratio of the extracted common factors is not less than 40% [34]. In the EFA model, the Brief**-**ERBS had three common factors, which were in accordance with the American version. In the American version, the three extracted factors accounted for only 36.00% of the total variance, and the three-factors CFA model had acceptable model fit (CMIN/DF = 1.630, CFI = 0.930, RMSEA = 0.050) [28]. However, in the Brief**-**ERBS, all item loads in the common factors were above 0.40, the communality of each item ranged between 0.359 and 0.680, and the total accounted for variance was 51.023%, which were highly interpretable and demonstrated excellent construct validity [33]. In the CFA, the model fit indices were evaluated by CMIN/DF < 5, GFI > 0.900, AGFI > 0.900, CFI > 0.900, IFI > 0.900, TLI > 0.900, RMSEA < 0.08 [35]. The CFA showed all measurements of the model are well fitted in our research, CMIN/DF = 4.092, GFI = 0.949, AGFI = 0.927, CFI = 0.913, IFI = 0.914, TLI = 0.908, RMSEA = 0.061. The results indicated that there is strong factor loading and interpretation variance, accord with EFA results, and have an excellent model fitting index.

For the discriminant validity, the scores of the top and low groups reached the level of significance (*P* < 0.001). Therefore, the discriminant validity was excellent.

As for convergent validity, a highly positive correlation between the Brief-ERBS total score and the ERBS total score was found (*r* = 0.972, *P* < 0.01), which indicate that the Brief-ERBS has excellent convergent validity.

The recommended value of Cronbach’s α coefficient is not lower than 0.60 [33]. In the Brief**-**ERBS, the overall Cronbach’s α coefficient was 0.798, which indicate acceptable homogeneity. The Cronbach’s α coefficient was 0.782 in the dimension of Regulation Worth, which was slightly higher than the original version. In other two dimensions, the Cronbach’s α coefficient was all somewhat less than the American version. As the number of items decreased, internal consistency decreased. The split-half reliability was 0.784. Moreover, the item-total score correlations were statistically significant, and the correlations ranged from 0.400 and 0.634, within the recommended standard (not smaller 0.4) [33]. Therefore, the homogeneity of the Brief**-**ERBS was acceptable. The retest coefficient was 0.879, which indicated acceptable stability.

In general, the results showed that the Brief**-**ERBS had good content validity, construct validity, discriminant validity, convergent validity, as well as homogeneity and stability. Therefore, the Brief**-**ERBS is a suitable tool for assessing Emotion and Regulation for nursing students. Furthermore, measuring the emotions may be culturally sensitive in some culture. However, emotions and regulation belief are vital psychological constructs that need to be measured as well as possible, so that researchers can well understand the impact on psychological processes, and timely intervention can be provided based on the assessment [36]. In order to increase effective response rate, the emotions and regulation belief can be collected by interviewer administered such as anonymous way and web-based surveys [37, 38]. In our study, the anonymous measures were taken and most of the participants finished the questionnaires effectively.

## Limitations

A cross-sectional study was conducted in our study, so further work is needed with longitudinal research to confirm these results. The participants were nursing students in two universities of Liaoning Province, and only two schools were selected in the survey. Hence, further work is needed to expand the sample coverage and take into consideration the adaptability of different groups. However, as a result of our study, a brief self-assessment tool is available for Chinese nursing undergraduate students to evaluate Emotion and Regulation belief.

## Conclusion

The study examined the psychometric properties of the Brief-ERBS in Chinese nursing students and showed good validity and reliability of the scale. The content and structure are simple, the evaluation method is flexible, and may be used for the beliefs about emotional management in Chinese nursing students.

## Supplementary Information

**Additional file 1.** 

## Data Availability

The datasets generated and/or analysed during the current study are not publicly available to preserve anonymity of the respondents but are available from the corresponding author on reasonable request.

## Abbreviations

ERBS: Emotion and Regulation Beliefs Scale
Brief-ERBS: The Chinese version of Emotion and Regulation Beliefs Scale consisting of 14 items
CVI: Content validity index
EFA: Exploratory factor analysis
CFA: Confirmatory factor analysis
KMO: Kaiser–Meyer–Olkin
CMIN/DF: Chi-square/degree of freedom
GFI: Goodness of fit index
AGFI: Adjusted goodness of fit index
CFI: Comparative fit index
IFI: Incremental fit index
TLI: Tucker Lewis index
RMSEA: Root-mean-square error of approximation
