# Supplementary material for: Reliability and validity of the Chinese version of the Brief Emotion and Regulation Beliefs Scale in Chinese nursing students
Source: BMC Nurs. 2022 Aug 9;21:221. doi: 10.1186/s12912-022-00992-1 (PMC9364589; doi:10.1186/s12912-022-00992-1)
Supplement: Supplementary file 1 — Additional file 1. [file 12912_2022_992_MOESM1_ESM.docx]

Emotion regulation processes are goal-oriented behaviors functioning to modify dynamic features of emotion, such as the magnitude and duration of behavioral, experiential, and physiological responses^[^[^1^](#_ENREF_1)^,^ [^2^](#_ENREF_2)^]^. Emotion regulation refers to the processes by which we influence which emotions we have, when we have them, and how we experience and express them. It could be affected by intra- and extra-familial social factors ^[^[^3^](#_ENREF_3)^]^, and also improved by interventions^[^[^4^](#_ENREF_4)^]^. Emotion dysregulation has been linked to a variety of mental problems ^[^[^5^](#_ENREF_5)^,^ [^6^](#_ENREF_6)^]^, and teens with difficulty modulating emotions may be involved in more illegal activities, for poorly regulated emotions may interfere with cognitive function that reminds youth of rules during decision making^[^[^7^](#_ENREF_7)^]^. Conversely, effective emotion regulation promotes mental health and is related to multiple positive mental outcomes, such as greater perceived well-being, better interpersonal relationships, and better physical health^[^[^5^](#_ENREF_5)^]^. As a result, emotion regulation is crucial to successful social interactions and health and is used a lot in our daily life^[^[^8-10^](#_ENREF_8)^]^.

Nursing students are facing a range of pressure, such as academic pressure, interpersonal pressure, and professional pressure, which cause emotional issues easily. Nursing education involves situations where students engage in interaction with other people, learning to care for and help patients involves a variety of emotions^[^[^11^](#_ENREF_11)^]^. For example, dissection and autopsies in the course of the preclinical study can provoke strong emotional reactions in some medical students^[^[^12^](#_ENREF_12)^]^. The clinical placement experiences can elicit negative emotions in nursing students. However, nursing students may be unprepared for regulating their emotions ^[^[^13-15^](#_ENREF_13)^]^. Emotion is a key source of stress for the early career of nurses and nursing students. Developing emotional intelligence behaviors should be a very useful measure to improve academic and clinical performance^[^[^16-18^](#_ENREF_16)^]^, and effective emotion regulation to enhance nursing students’ professional identity and build the skills for effective patient care and their health and well-being^[^[^19^](#_ENREF_19)^,^ [^20^](#_ENREF_20)^]^. In clinical work, nurses do a lot of physical and mental work, but also pay more emotional work^[^[^21^](#_ENREF_21)^,^ [^22^](#_ENREF_22)^]^. In recent years, more and more scholars have realized the importance of nurses' physical and mental health to guarantee clinical nursing quality and stabilize nursing teams^[^[^21^](#_ENREF_21)^,^ [^23^](#_ENREF_23)^,^ [^24^](#_ENREF_24)^]^. As future nurses, nursing students are experiencing complex learning environments and will experience complex work environments in future clinical work, which probably lead to emotional problems directly^[^[^25^](#_ENREF_25)^,^ [^26^](#_ENREF_26)^]^. However, one’s beliefs about controlling their emotions portend a series of vital psychological outcomes^[^[^27^](#_ENREF_27)^]^. So it is especially important to search for suitable tools to assess the emotion and regulation beliefs of nursing students, and give timely intervention to improve their physical and mental health^[^[^10^](#_ENREF_10)^,^ [^27^](#_ENREF_27)^]^.

The Emotion and Regulation Beliefs Scale (ERBS), which was originally developed by Veilleux in America, is a simple and effective tool to assess emotion and regulation belief. The EBRS assesses beliefs that emotions can hijack self-control, emotion regulation is a worthwhile pursuit, and emotions can constrain behavior. The ERBS has excellent internal consistency and powerfully predicts clinically relevant outcomes even after controlling for an existing short measure of beliefs in emotion controllability^[^[^28^](#_ENREF_28)^]^.

Therefore, this study was to translate the American version of the ERBS into simplified Chinese and to test the validity and reliability of the Chinese version of ERBS in nursing students.

The study examines the psychometric properties of the ERBS (Chinese version) in Chinese nursing students and shows good validity and reliability of the scale, the content and structure are simple, the evaluation method is flexible, and may be used for the beliefs about emotional management in Chinese nursing students.

1. Thompson RA: **Emotion regulation: a theme in search of definition**. *Monogr Soc Res Child Dev* 1994, **59**(2-3):25-52.

2. Christiansen H, Hirsch O, Albrecht B, Chavanon ML: **Attention-Deficit/Hyperactivity Disorder (ADHD) and Emotion Regulation Over the Life Span**. *Current psychiatry reports* 2019, **21**(3):17.

3. Cole PM, Ashana Ramsook K, Ram N: **Emotion dysregulation as a dynamic process**. *Dev Psychopathol* 2019, **31**(3):1191-1201.

4. Andreescu C, Sheu LK, Tudorascu D, Gross JJ, Walker S, Banihashemi L, Aizenstein H: **Emotion reactivity and regulation in late-life generalized anxiety disorder: functional connectivity at baseline and post-treatment**. *Am J Geriatr Psychiatry* 2015, **23**(2):200-214.

5. Kneeland ET, Dovidio JF, Joormann J, Clark MS: **Emotion malleability beliefs, emotion regulation, and psychopathology: Integrating affective and clinical science**. *Clin Psychol Rev* 2016, **45**:81-88.

6. Calkins SD, Dollar JM, Wideman L: **Temperamental vulnerability to emotion dysregulation and risk for mental and physical health challenges**. *Dev Psychopathol* 2019, **31**(3):957-970.

7. Kemp K, Thamotharan S, Poindexter B, Barker D, Tolou-Shams M, Houck CD: **EMOTION REGULATION AS A PREDICTOR OF JUVENILE ARREST**. *Criminal justice and behavior* 2017, **44**(7):912-926.

8. Gyurak A, Goodkind MS, Kramer JH, Miller BL, Levenson RW: **Executive functions and the down-regulation and up-regulation of emotion**. *Cogn Emot* 2012, **26**(1):103-118.

9. Gross JJ: **Emotion regulation: taking stock and moving forward**. *Emotion* 2013, **13**(3):359-365.

10. DeSteno D, Gross JJ, Kubzansky L: **Affective science and health: the importance of emotion and emotion regulation**. *Health Psychol* 2013, **32**(5):474-486.

11. Weurlander M, Lonn A, Seeberger A, Broberger E, Hult H, Wernerson A: **How do medical and nursing students experience emotional challenges during clinical placements?** *Int J Med Educ* 2018, **9**:74-82.

12. Tseng WT, Lin YP: **"Detached concern" of medical students in a cadaver dissection course: A phenomenological study**. *Anatomical sciences education* 2016, **9**(3):265-271.

13. McCloughen A, Levy D, Johnson A, Nguyen H, McKenzie H: **Nursing students' socialisation to emotion management during early clinical placement experiences: A qualitative study**. *J Clin Nurs* 2020, **29**(13-14):2508-2520.

14. Edo-Gual M, Tomás-Sábado J, Bardallo-Porras D, Monforte-Royo C: **The impact of death and dying on nursing students: an explanatory model**. *J Clin Nurs* 2014, **23**(23-24):3501-3512.

15. Jack K, Wibberley C: **The meaning of emotion work to student nurses: a Heideggerian analysis**. *International journal of nursing studies* 2014, **51**(6):900-907.

16. Birks Y, McKendree J, Watt I: **Emotional intelligence and perceived stress in healthcare students: a multi-institutional, multi-professional survey**. *BMC Med Educ* 2009, **9**:61.

17. Cherry MG, Fletcher I, O'Sullivan H, Dornan T: **Emotional intelligence in medical education: a critical review**. *Med Educ* 2014, **48**(5):468-478.

18. McCloughen A, Foster K: **Nursing and pharmacy students' use of emotionally intelligent behaviours to manage challenging interpersonal situations with staff during clinical placement: A qualitative study**. *J Clin Nurs* 2018, **27**(13-14):2699-2709.

19. Lewis GM, Neville C, Ashkanasy NM: **Emotional intelligence and affective events in nurse education: A narrative review**. *Nurse Educ Today* 2017, **53**:34-40.

20. Saedpanah D, Salehi S, Moghaddam LF: **The Effect of Emotion Regulation Training on Occupational Stress of Critical Care Nurses**. *J Clin Diagn Res* 2016, **10**(12):Vc01-vc04.

21. Foster K, Roche M, Giandinoto JA: **Workplace stressors, psychological well-being, resilience, and caring behaviours of mental health nurses: A descriptive correlational study**. 2020, **29**(1):56-68.

22. Rouxel G, Michinov E, Dodeler V: **The influence of work characteristics, emotional display rules and affectivity on burnout and job satisfaction: A survey among geriatric care workers**. *Int J Nurs Stud* 2016, **62**:81-89.

23. Peng YN, Liu CY, Zhang H, Zhang N, Cao Y, Wu YY: **[Psychological health of nurses in operating room participating in organ donation and intervention effect]**. *Zhonghua Lao Dong Wei Sheng Zhi Ye Bing Za Zhi* 2020, **38**(4):271-274.

24. Glasofer A, Townsend AB: **Supporting nurses' mental health during the pandemic**. *Nursing* 2020, **50**(10):60-63.

25. Sun N, Wei L, Shi S, Jiao D, Song R, Ma L, Wang H, Wang C, Wang Z, You Y *et al*: **A qualitative study on the psychological experience of caregivers of COVID-19 patients**. *American journal of infection control* 2020, **48**(6):592-598.

26. Khalaf IA, Al-Dweik G, Abu-Snieneh H, Al-Daken L, Musallam RM, BaniYounis M, Al-Rimawi R, Khatib AH, Habeeb Allah A, Atoum MH *et al*: **Nurses' Experiences of Grief Following Patient Death: A Qualitative Approach**. *J Holist Nurs* 2018, **36**(3):228-240.

27. De Castella K, Platow MJ, Tamir M, Gross JJ: **Beliefs about emotion: implications for avoidance-based emotion regulation and psychological health**. *Cogn Emot* 2018, **32**(4):773-795.

28. Veilleux JC, Salomaa AC, Shaver JA, Zielinski MJ, Pollert GA: **Multidimensional assessment of beliefs about emotion: development and validation of the emotion and regulation beliefs scale**. *Assessment* 2015, **22**(1):86-100.
